# Improvement of Light Output of MAPbBr_3_ Single Crystal for Ultrafast and Bright Cryogenic Scintillator

**DOI:** 10.1021/acs.jpclett.4c00379

**Published:** 2024-03-28

**Authors:** Somnath Mahato, Michal Makowski, Shaona Bose, Dominik Kowal, Md Abdul Kuddus Sheikh, Philipp Braueninger-Wemer, Marcin E. Witkowski, Samit Kumar Ray, Winicjusz Drozdowski, Muhammad Danang Birowosuto

**Affiliations:** † Lukasiewicz Research Network - PORT Polish Center for Technology Development, Wroclaw 54-066, Poland; ‡ Department of Physics, Indian Institute of Technology Kharagpur, Kharagpur-721 302, India; § Cintilight LLC, Knoxville, Tennessee 37919, United States; ∥ Institute of Physics, Faculty of Physics, Astronomy, and Informatics, Nicolaus Copernicus University in Torun, Torun 87-100, Poland

## Abstract

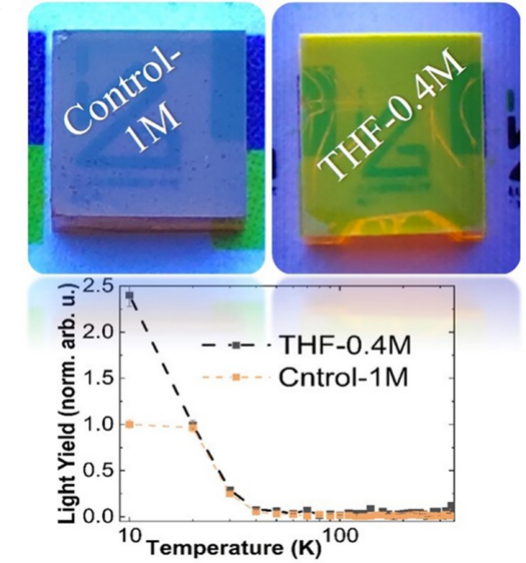

The remarkable brightness and rapid scintillation observed in perovskite single crystals (SCs) become even more striking when they are operated at cryogenic temperatures. In this study, we present advancements in enhancing the scintillation properties of methylammonium lead bromide (MAPbBr_3_) SCs by optimizing the synthesis process. We successfully synthesized millimeter-sized MAPbBr_3_ SCs with bright green luminescence under UV light. However, both MAPbBr_3_ (Control-1M and THF-0.4M) SCs display notable radioluminescence exclusively at low temperatures due to their phase transitions. Notably, the THF-0.4M SCs exhibit a remarkable improvement in radioluminescence light yield, surpassing Control-1M SCs more than 2-fold. Further, THF-0.4M SCs demonstrate an ultrafast decay component of 0.52 ns (82.2%) and a slower component of 1.80 ns (17.8%), contributing to a rapid scintillation response at low temperatures. Therefore, the amalgamation of ultrafast decay components and improved radioluminescence light yield equips THF-0.4M SCs to emerge as a top choice for perovskite scintillators for X-ray timing applications.

Organic–inorganic lead halide perovskites, MAPbX_3_ (X = I, Br, Cl), have attracted a lot of attention in recent years due to potential applications such as in optoelectronic devices, radiation detectors, and scintillators.^1−5^ Scintillators can convert X-ray photons into UV/vis photons in the form of electrons via the photoelectric effect and play a significant role in developing X-ray imaging and scintillation detectors. While several types of perovskite materials^6,7^ have been used as X-ray scintillators, there are still many issues and limitations for their commercialization.^8,9^ Therefore, the exploration of affordable, high-performance scintillation materials remains a significant focus of both scientific inquiry and practical application.

Recently, hybrid perovskite single crystals (SCs) have demonstrated direct X-ray imaging due to their strong X-ray absorption and efficient conversion to charge carriers.^4^ In addition, two-dimensional (2D)^10−12^ and three-dimensional (3D)^13−15^ lead halide perovskite SCs have attracted significant attention as scintillators due to their ambient stability and chemical compositions, which exhibit high light yield and nanosecond decay times, making them ideal for use in the time-of-flight positron emission tomography (TOF-PET) technology.^16,17^ Cala’ et al.^18^ reported a light yield of 17,300 ph/MeV in undoped 2D PEA_2_PbBr_4_ SCs and its improvement up to 21,400 ph/MeV when doped with lithium at room temperature. However, Mykhaylyk et al.^14^ obtained maximum light yield in 3D CsPbBr_3_ SCs of 109,000 ph/MeV at 7 K and recently reported ultrafast scintillation response in CsPbCl_3_ SCs at low temperature with a light yield of 19,000 ph/MeV.^19^ Interestingly, Birowosuto et al.^13^ proposed the light yield theoretically can be in excess of 150,000 ph/MeV in 3D MAPbBr_3_ SCs at *T* = 10 K. Therefore, our investigation’s purpose is to assess the feasibility of using perovskite SCs as cryogenic scintillation detectors by examining and analyzing their scintillation properties at varying temperatures. Here we focus on 3D methylammonium lead bromide (MAPbBr_3_) SCs, a subset of the perovskite SCs family that has gained considerable interest over the past few decades. Recently, we made significant and moderate improvements to the surface emission, quantum yield, and conductivity of the crystals, respectively.^20^ Now we have discovered that the two latter improvements also enhance the light yield (by 2 times) and decrease the decay time (to less than 1 ns) at 10 K. This ultrafast decay time positions MAPbBr_3_ SCs among the fastest scintillators, surpassing other perovskite SCs in terms of decay components.

Several crystallization techniques such as antisolvent vapor-assisted crystallization (AVC), top-seeded growth, liquid-diffused separation-induced crystallization, and inverse temperature crystallization (ITC)^21^ have been reported for growing superior-quality MAPbBr_3_ SCs. Boopathi et al.^22^ developed a crystallization technique involving solvent acidolysis crystallization (SAC) that relied on the acidolysis of *N*-methylformamide (NMF) for in situ formation of the MA cation to grow MAPbBr_3_ perovskite SCs showing green edge emission under UV light. Gidey et al.^23^ reported in situ growth of a polycrystalline MAPbBr_3_ layer on the top surface of bulk millimeter-sized SCs by modifying the precursor solution concentration along with using dichloromethane, an antisolvent of dimethylformamide, to initiate the crystallization, and they obtained 5-fold improved sensitivity of X-ray detection. In our previous report, we optimized the concentration of antisolvent tetrahydrofuran (THF) to grow bigger-sized (≥5 mm) and high-quality MAPbBr_3_ perovskite SCs displaying bright emission from the entire top and side surface under UV light. Therefore, with these MAPbBr_3_ SCs with high crystalline quality, efficient bright emission, and low-cost solution processability, MAPbBr_3_ SCs can be used as bright and fast scintillators. However, there is a lack of reports on transparent and bright-green surface-emissive MAPbBr_3_ SCs for cryogenic scintillator applications, particularly ones exhibiting ultrafast decay times (e.g., timing applications, photon counting computed tomography, and time-of-flight X-ray imaging).^15,24,25^

In this study, we successfully grew high-quality transparent millimeter*-*sized MAPbBr_3_ SCs, resulting in a bright green emission from the surface upon exposure to UV light. The detailed synthesis process was described in the previous work.^20^ Cathodoluminescence (CL) images demonstrated that THF-0.4M crystals exhibited enhanced luminescence signals, and photoluminescence (PL) measurements revealed a 10-fold increase in PL intensity compared to Control-1M MAPbBr_3_ SCs. To explore the luminescent and scintillation properties of a MAPbBr_3_ (Control-1M and THF-0.4M) single crystal as a function of temperature, we used a 375 nm laser and X-rays. According to our low-temperature PL measurements, we have identified a structural phase transition (both SCs) from cubic to orthorhombic occurring at 50 K. However, the noticeable spectral difference in Control-1M crystals is broader compared to THF-0.4M crystals. Further, in the case of Control-1M, there is a slight, discernible acceleration in the thermal quenching of luminescence, and a substantial drop is observed at a temperature of 30 K, whereas for sample THF-0.4M, this quenching occurs within the range of 40–50 K. Notably, THF-0.4M SCs reveal a doubled radioluminescence light yield (RLY) in comparison with Control-1M SCs. Finally, the luminescence decay kinetics of both our crystals under pulsed X-ray excitation revealed an ultrafast decay behavior, with an effective decay time below 1 ns.

We optimized the method for crystallizing the quality of MAPbBr_3_ SCs by employing antisolvent-assisted solvent acidolysis at room temperature, specifically using an antisolvent. This optimized approach results in high-quality crystals that exhibit bright emission across the entire top surface when exposed to UV light, as shown in Figure 1. We compared this bright surface emission with the same MAPbBr_3_ SCs synthesized by conventional inverse temperature crystallization techniques^21^ (Control-1M), which are entirely nonemissive under UV light. The detailed synthesis process of THF-0.4M and Control-1M crystals is described in the Supporting Information (SI). The crystallographic structures of Control-1M and THF-0.4M MAPbBr_3_ SCs were investigated with X-ray diffraction (XRD). The XRD measurement of the upper facet of both MAPbBr_3_ SCs (shown in Figure S1) indicates regularly and periodically repeated diffraction peaks along the (001) plane, the normal direction of cubic-phase MAPbBr_3_ crystals. The sharp and intense peaks in THF-0.4M crystals suggest a superior quality of single-crystalline MAPbBr_3_ compared to that of Control-1M crystals.

**Figure 1:**
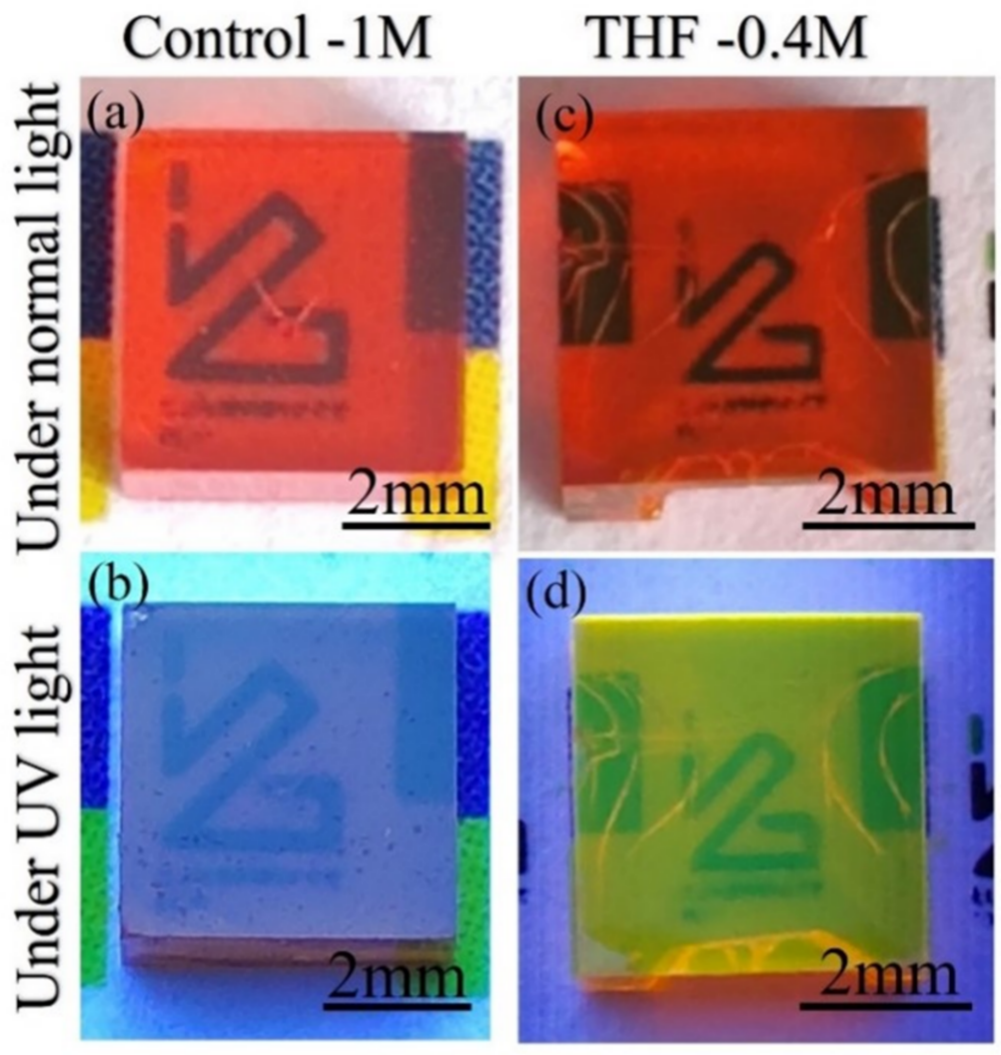
Photographs of (a, b) Control-1M and (c, d) THF-0.4M MAPbBr_3_ single crystals under normal light and UV light.

To further investigate the surface emission of both SCs (Control-1M and THF-0.4M), we conducted CL and PL analyses at room temperature. The THF-0.4M crystals demonstrated significantly stronger CL emission compared with the Control-1M crystals. Further, we examined PL and time-resolved PL (TRPL) spectra using the same excitation power and spectrometer integration time on the surface of the Control-1M and THF-0.4M MAPbBr_3_ SCs (Figure 2). The PL of the THF-0.4M crystal (emissive crystals) is 10 times more intense than that of the Control-1M crystal (nonemissive crystals). Also, the PL spectrum of the emissive surface is red-shifted about 5 nm with respect to that of the nonemissive crystals. However, although the PL intensity from the surface is significantly enhanced, the quantum yield of the bulk THF-0.4M crystal only improves 2-fold from 3% for the Control-1M crystal to 6%.^20^ The measured PL decay curves (Figure 2 inset) show triple-exponential behaviors. The average decay time of the emissive crystals (ECs) was obtained as τ^EC^_av_ = 55.6 ± 0.3 ns with components τ^EC^_1_ = 0.5 ± 0.01 ns (2%), τ^EC^_2_ = 5.5 ± 0.02 ns (12%), and τ^EC^_3_ = 63.6 ± 0.3 ns (86%). The average decay time of the nonemissive crystals (NECs) is τ^NEC^_av_ = 29.6 ± 0.4 ns, which is substantially shorter than for the emissive one, and the three decay constants are τ^NEC^_1_ = 0.6 ± 0.01 ns (7%), τ^NEC^_2_ = 6 ± 0.02 ns (38%), and τ^NEC^_3_ = 50 ± 0.6 ns (55%), Therefore, the THF-0.4M crystals exhibit a significantly longer PL mean decay time than Control-1M crystals.

**Figure 2:**
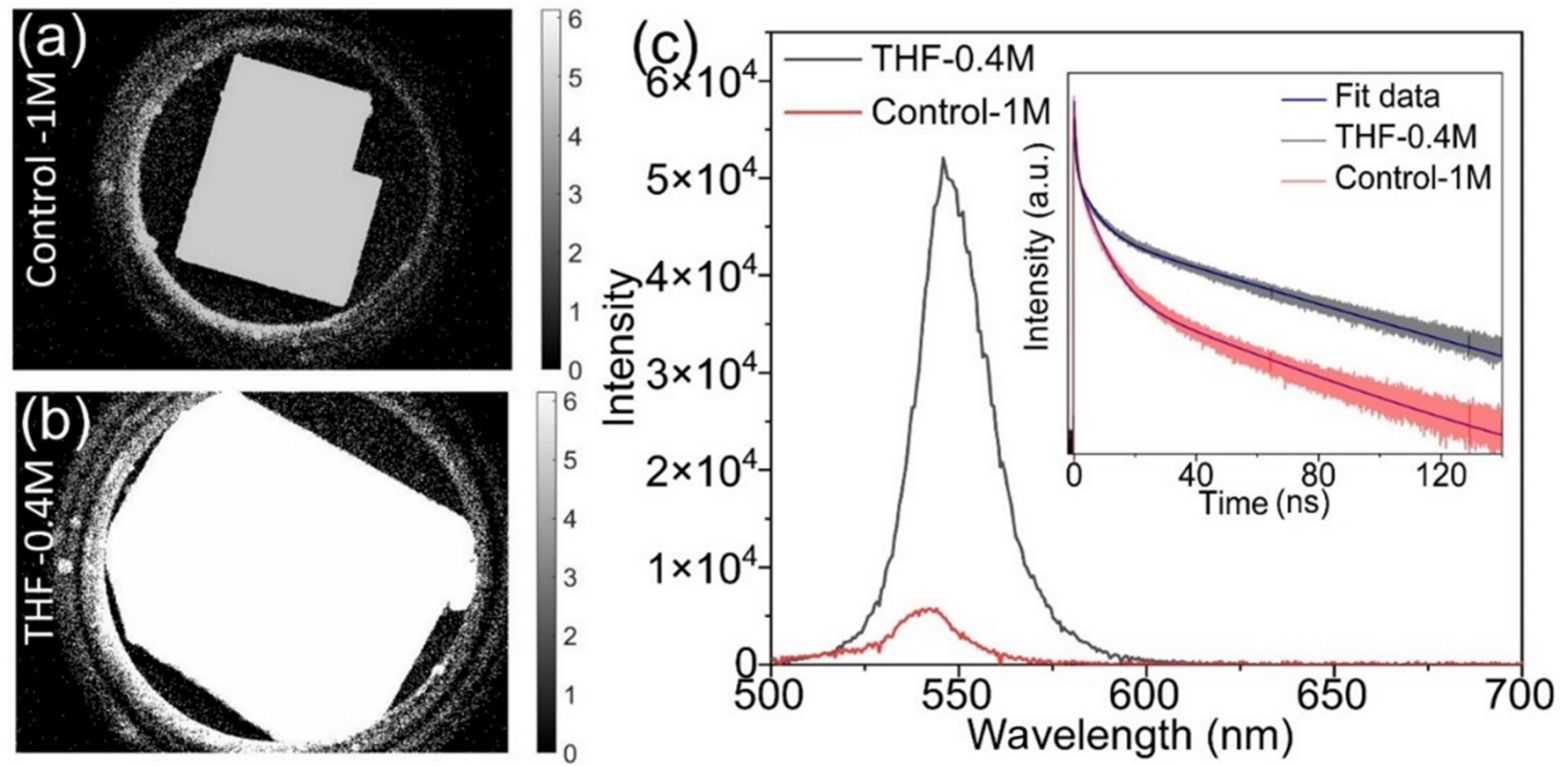
(a, b) Cathodoluminescence images of the top surfaces of (a) Control-1M and (a) THF-0.4M MAPbBr_3_ SCs. (c) Room-temperature photoluminescence (PL) and time-resolved photoluminescence (TRPL) of the same Control-1M and THF-0.4M MAPbBr_3_ SCs.

The analysis of the crystal structure at room temperature using XRD data validates the cubic phase (*Pm*3*m*) of MAPbBr_3_, for both synthesized SCs as discussed earlier. However, the structural phase transitions from cubic to tetragonal to orthorhombic phase in MAPbBr_3_ single crystals were observed by Liang et al.^26^ and Abia et al.^27^ during low-temperature XRD measurements. Therefore, study of the phase transitions plays a significant role in understanding the thermoluminescence/radioluminescence properties of both MAPbBr_3_ single crystals. Here temperature-dependent PL measurements were carried out from 300 to 10 K, and the results are shown in Figure 3. As the temperature goes down to approximately 50 K, the PL peak shifts slightly to the lower-energy side and jumps from 2.33 to 2.22 eV and from 2.29 to 2.16 eV for Control-1M and THF-0.4M crystals, respectively, where the crystal phase of both MAPbBr_3_ SCs changes from cubic to orthorhombic.^28^

**Figure 3:**
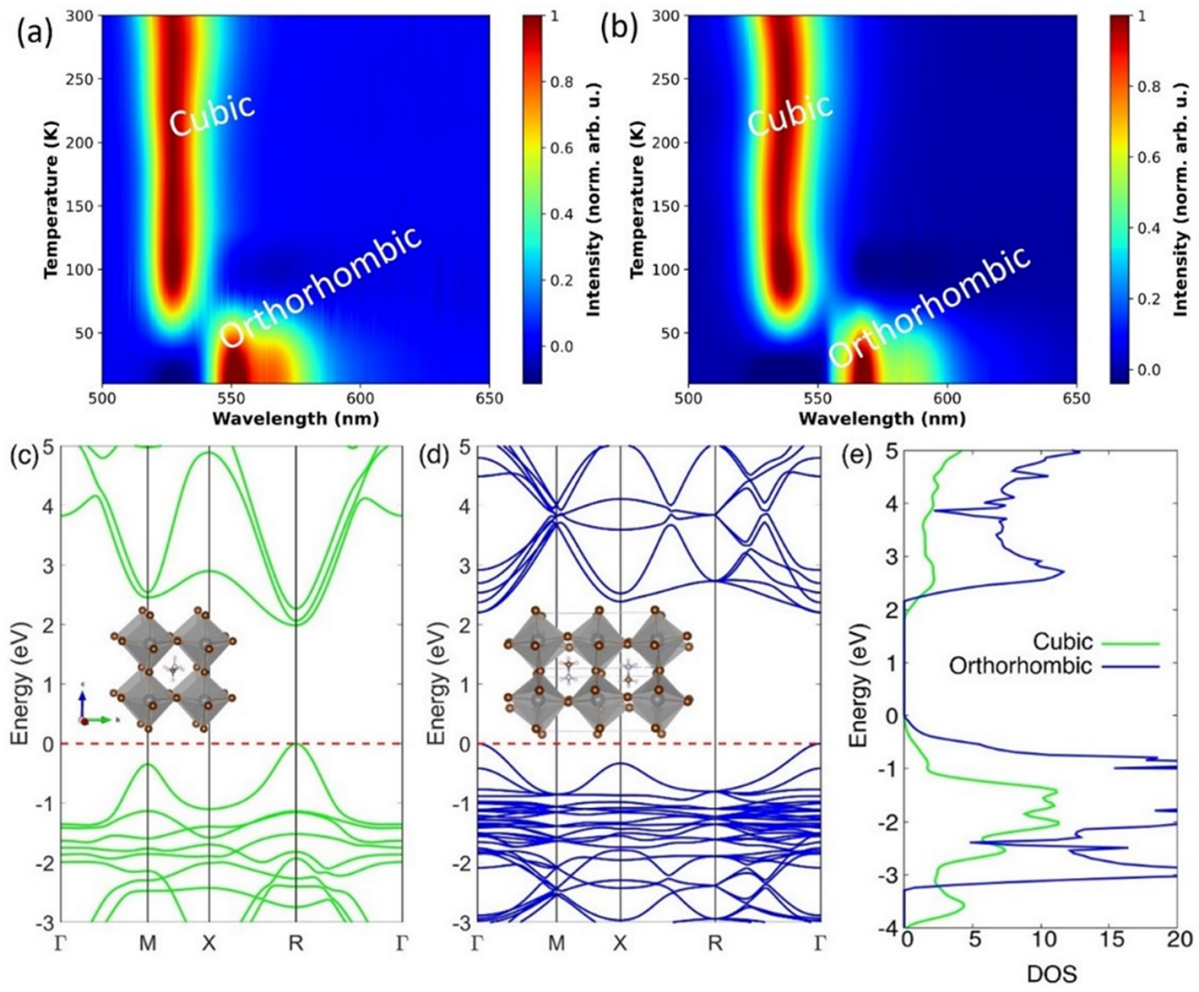
(a, b) Low-temperature photoluminescence of (a) Control-1M and (b) THF-0.4M MAPbBr_3_ single crystals. (c, d) Band structures of (c) cubic and (d) orthorhombic MAPbBr_3_ with their respective atomic structures shown in the insets. (e) DOS of both phases of MAPbBr_3_.

Interestingly, our DFT calculations also predict a denser band structure for the orthorhombic phase compared to the cubic phase. The energy of cubic MaPbBr_3_ (Figure 3c inset) computed from first-principles calculations as implemented in Quantum Espresso, was −1537.798 Ry, and that of orthorhombic MAPbBr_3_ (Figure 3d inset) was found to be −6151.231 Ry. Hence, the ground-state energy of orthorhombic MAPbBr_3_ is greater than that of its cubic counterpart by −0.13 eV per formula unit, which causes the orthorhombic phase to be more stable at lower temperatures. The greater number of atoms per unit cell in the orthorhombic phase results in a band structure that is denser than the cubic phase band structure, as shown in Figure 3c,d. A closer look at the density of states (DOS) plot in Figure 3e indicates a higher availability of electron energy states close to the valence and conduction bands that facilitates more frequent band transitions of electrons in the orthorhombic phase.

Radioluminescence spectral maps and thermal quenching curves of Control-1M and THF-0.4M are shown in Figure 4a,b. Upon careful examination, it becomes evident that there is an almost negligible signal detected at temperatures surpassing 50 K. Surprisingly, the phase transition also occurs at that temperature, see Figure 3a,b. This observation leads to the deduction that the application of the MAPbBr_3_ perovskite is exclusively viable within the domain of cryogenic scintillation applications. This conclusion resonates with data previously documented and reported in the existing literature^13,14,19^ yet stands in a slight contradiction to Mykhaylyk’s observation,^15^ wherein visible emission was evident even at temperatures close to 200 K. Notably, the radioluminescence signal shown by the Control-1M crystals appears broader than that of the THF-0.4M crystals, with most of the intensity concentrated around 560 nm. Both crystals exhibit typical excitonic luminescence behavior with strong thermal quenching.^29^ In the orthorhombic phase of MAPbBr_3_, the predominance of free excitons is primarily attributed to the inorganic component (PbBr_2_), with minimal influence from the organic constituents. This phase exhibits a higher binding energy compared with the cubic or tetragonal phases, resulting in reduced luminescence quenching. The integrated areas under the RL curves were normalized and presented for quantitative analysis by Arrhenius fitting. Since they are two phases (below and above 50 K), we used a modified Arrhenius fit as shown below:
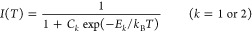
1where the symbols *E*_1_ and *E*_2_ denote the activation energies related to thermal quenching processes linked with nonradiative recombination, and *C*_1_ and *C*_2_ represent the ratios between the thermal quenching rate at temperature *T* = ∞ and the rates of radiative transition for temperatures between 10 and 50 K and between 50 and 350 K, respectively. A comprehensive compilation of the fitted parameters is presented in Table 1. It is evident from the table that the significant changes in the quenching for both samples happen for temperatures below 50 K (index 1). For this range, the activation energy *E*_1_ associated with nonradiative recombination processes decreases from almost 20 to less than 8 meV when transitioning from Control-1M to THF-0.4M crystal. Although *E*_1_ of the THF-0.4M crystal is almost 2.5 times smaller than *E*_1_ of the Control-1M crystal (likely to have more quenching by the activation of the electrons to the conduction band), the ratio *C*_1_ of the Control-1M crystal is more than 40.8 times larger than that of the THF-0.4M crystal, and thus, other processes may contribute to the thermal quenching.^30^ Upon meticulous analysis of low-temperature glow curves (Figure S3), the outline of a trap peak becomes visible within the THF-0.4M crystals, reaching its apex at approximately 50 K. Nevertheless, due to an exceedingly poor signal-to-noise ratio (negligibly low intensity of the observed peak), it is impossible to accurately fit it to ascertain its defining parameters. Nonetheless, the presence of such a peak is noteworthy, and it is worth mentioning that its occurrence correlates with previously published data.^13^ However, in contrast to the previously reported observations,^13^ the analysis conducted through low-temperature thermoluminescence failed to reveal any evidence of trapping states within the energy gap of the Control-1M crystal. Moreover, the lack of afterglow serves as further evidence attesting to the exceptional quality of the developed crystals (Figure S4). Figure 4c shows the RLYs as a function of temperature. In principle, they are similar to the insets in Figure 4a,b, but now they were normalized with the LY obtained from the pulse height spectra at 10 K shown in Figure S5 (where 1.0 is the LY of the Control-1M crystal).^15,31^ Notably, a significant dissimilarity is observed solely at 10 K between the two crystals under examination. Specifically, the THF-0.4M crystal demonstrates an increase of 2.4 times in comparison to the Control-1M crystal. The increase in RLY at cryogenic temperatures is linked to the increase of the modest quantum yield (2 times) and conductivity (almost 2 times) of the bulk crystals.^20^ The latter is strongly related to the transfer efficiency in the scintillation mechanism process.^13^ Nevertheless, this enhancement of the RLY comes with a price of prolongation of the X-ray-induced decay time shown in Figure 5a. The brighter crystal reveals twice as long mean decay time as the Control-1M crystal, yet both reveal an ultrafast decay behavior of 0.72 and 0.45 ns for THF-0.4M and Control-1M crystals, respectively. To facilitate quantitative analysis of the slower decay times, we measured the reflectance (*R*) and transmittance (*T*) data, meticulously corrected by the sensitivity of the measurement, for both the Control-1M and THF-0.4M crystals, as shown in Figures S6 and S7. The THF-0.4M crystals exhibit higher reflectance compared to the Control-1M crystals, which contributes to the increase of brightness at room temperature. Furthermore, the narrower reflectance in THF-0.4M crystals in comparison to transmittance suggests reduced overlap, implying lower self-absorption at lower temperatures compared to Control-1M crystals. In contrast, the Control-1M sample displays no discernible reflectance or transmittance at RT, indicating significant self-absorption effects. At lower temperatures, there is a notably stronger reflectance; however, the increased overlap with transmittance suggests higher self-absorption.

**Figure 4:**
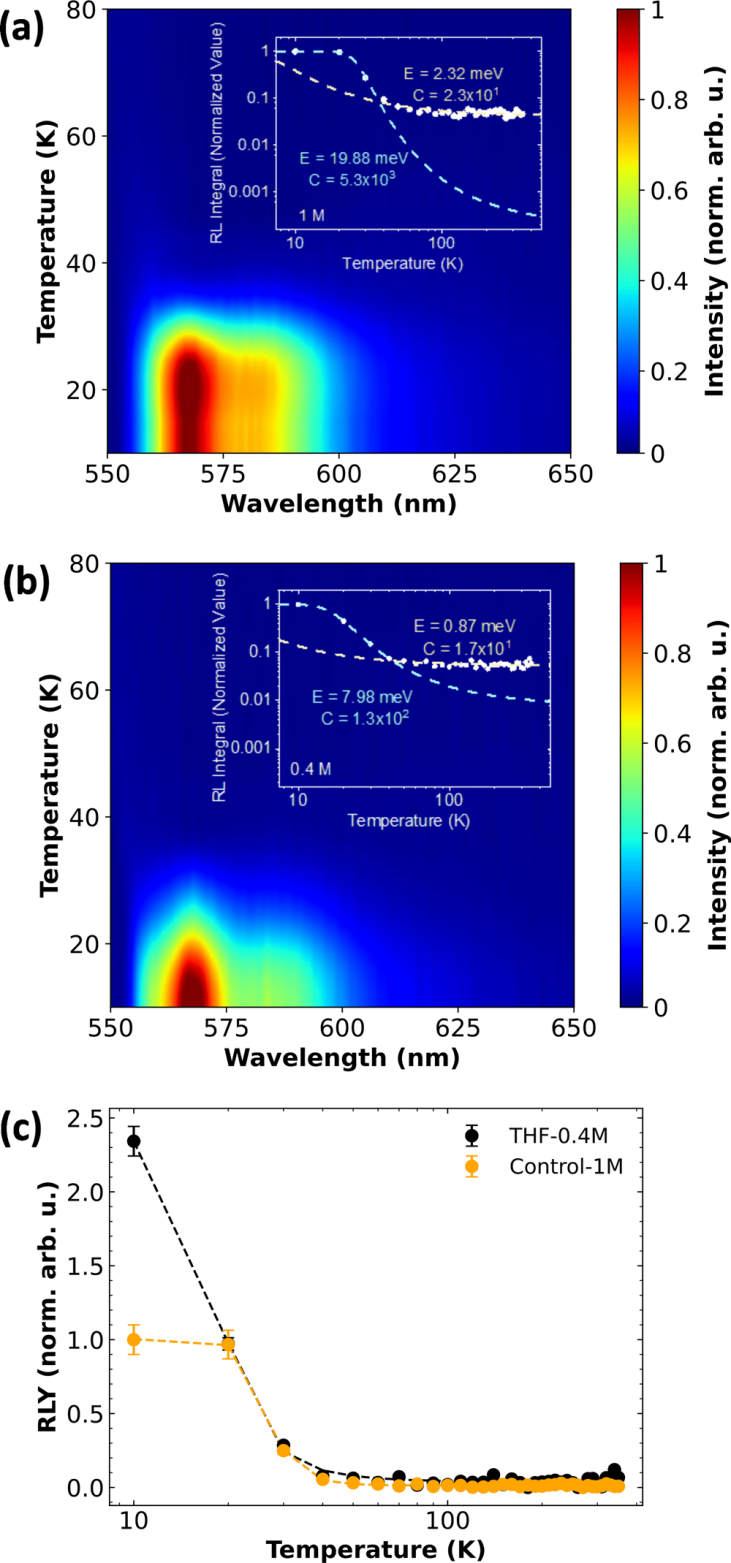
(a, b) Radioluminescence maps of (a) Control-1M and (b) THF-0.4M MAPbBr_3_ single crystals. The insets present integrated RL curves for a whole temperature range with applied Arrhenius fitting, divided into two regions, 10–50 K and 50–350 K. (c) Radioluminescence light yields as a function of temperature.

**Figure 5:**
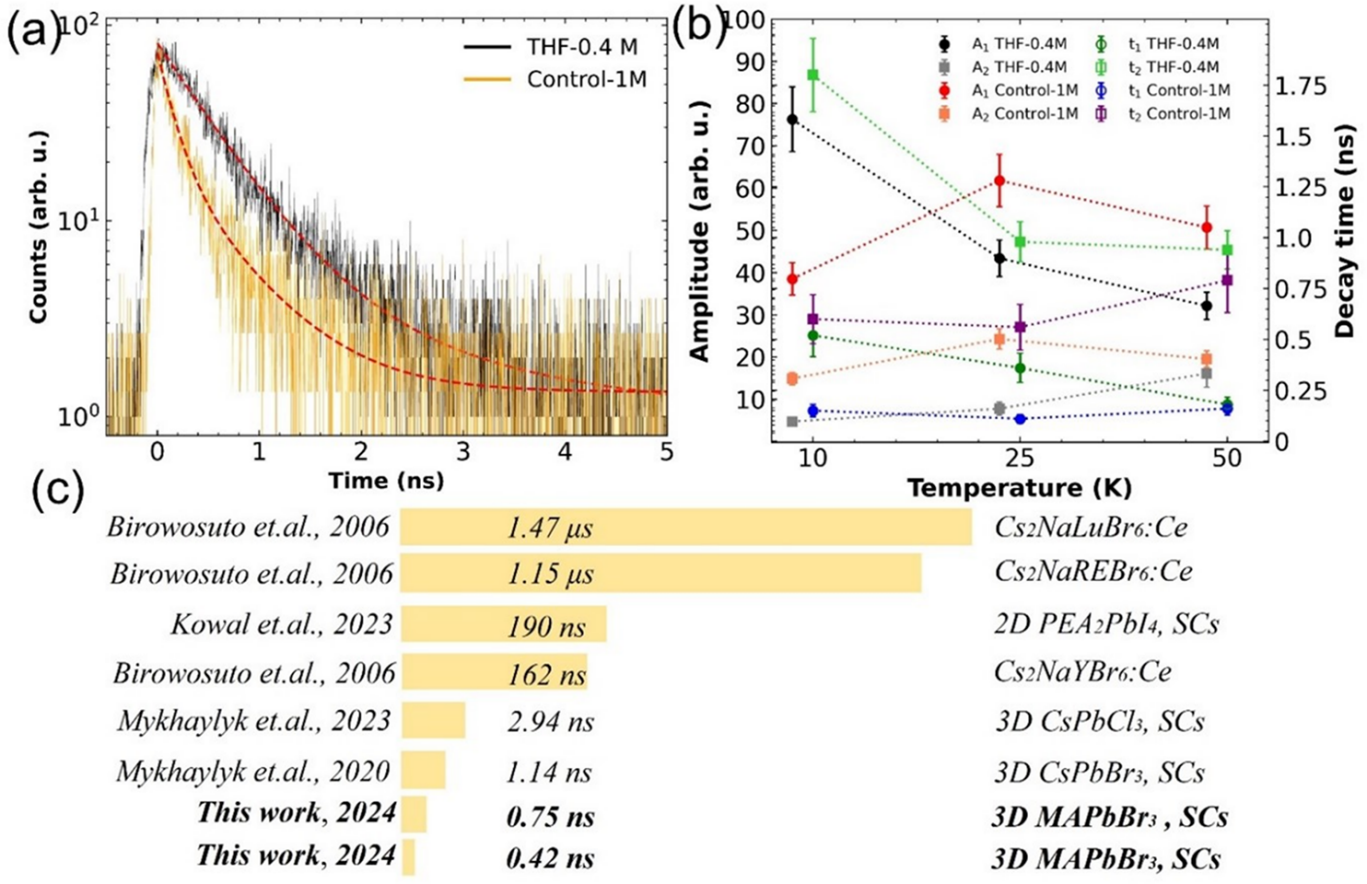
(a) X-ray-induced luminescence decay. (b) X-ray-excited decay fit parameters at 10, 25, and 50 K. Relative decay time observed in perovskite and other single crystals at low temperature reported in the literature, including the results reported by Birowosuto et al.,^32^ Kowal et al.,^10^ Mykhaylyk et al.^14,19^ and this work.

**Table 1:** Comparison of Scintillation Properties of THF-0.4M and Control-1M Single Crystals

	10–50 K		50–350 K		10 K		
MAPbBr_3_	*E*_1_ (meV)	*C*_1_	*E*_2_ (meV)	*C*_2_	X-ray decay (ns)	τ_d_	RLY
THF-0.4M	7.98	1.3 × 10^2^	0.87	1.7 × 10^1^	0.52 ± 0.01 (82.2%)	0.75 ± 0.01	1.71
					1.80 ± 0.21 (17.8%)		
Control-1M	19.88	5.3 × 10^3^	2.32	2.3 × 10^1^	0.15 ± 0.01 (39.7%)	0.42 ± 0.01	0.71
					0.60 ± 0.03 (60.3%)		

Finally, Figure 5b presents an analysis of X-ray decay curves measured at 10, 25, and 50 K and fitting parameters of the double-exponential function. The amplitude variations exhibit a degree of symmetry between the components for individual samples. The THF-0.4M crystal exhibits a shortening of both time constants with an increase of temperature, while for Control-1M it is more stable. As a result, the scintillation mechanism in the crystals at low temperatures is governed by two primary processes, contributing to the fast and slow emission components. The fast decay component corresponds to the radiative decay of free excitons, while the slow component of the emission is ascribed to the radiative decay of electrons and holes liberated from the traps. By taking an effective decay time into consideration, it is visible that at 10 K both types of our perovskites are outperforming the competition significantly, e.g., MAPbBr_3_ τ_d_ = 47 ns^15^ or τ_d_ = 30.5 ns for LGSO-Ce.^28^

In summary, our investigation is focused on exploring the radioluminescence and scintillation properties of MAPbBr_3_ SCs across varying temperatures. We successfully optimized the growth process to produce high-quality, transparent MAPbBr_3_ SCs suitable for cryogenic scintillators. Notably, THF-0.4M single crystals exhibited a doubled radioluminescence light yield (RLY) compared to Control-1M single crystals. Moreover, an in-depth analysis of the decay kinetics of THF-0.4M SCs revealed an ultrafast decay component of 0.52 ns (82.2%) and a slower component of 1.80 ns (17.8%). This dual-component decay contributes to a rapid scintillation response at low temperatures. These findings strongly support the conclusion that THF-0.4M MAPbBr_3_ SCs are exceptionally well-suited for applications requiring a swift scintillation response at cryogenic temperatures. Such ultrafast scintillation decay times can be useful for X-ray imaging with timing applications,^25^ while the decay times still can be much improved through Purcell enhancements.^24,33^

## Experimental Section

*Photoluminescence and TRPL.* In a carefully controlled ambient environment at 300 K, temperature-dependent measurements were conducted meticulously over a range spanning 10 to 300 K. These measurements involved employing free space for both excitation and collection methodologies, facilitated through a visible–near-infrared microscope objective (Nikon 20×, NA = 0.40). PicoQuant’s pulsed lasers, emitting UV light at a 355 nm wavelength with a pulse width of 15 ps and a repetition rate of 10 MHz, were precisely directed onto the specimens using an Olympus microscope objective (40×, NA = 0.65), focusing the laser beam to an approximate diameter of 1 μm. The resulting PL spectra were meticulously captured by utilizing a thermoelectric-cooled Avaspec spectrometer. Following this, TRPL decay curves were derived meticulously by employing a 375 nm laser operating at a repetition rate of 200 kHz in tandem with a single-photonavigation photodiode (APD). The timing intricacies were rigorously analyzed by using sophisticated time-correlated single photon counting electronics (HydraHarp 400, PicoQuant, Germany)

*Radio- and Thermoluminescence.* Our experimental approach involved the utilization of a unified configuration accommodating both radioluminescence (RL) and thermoluminescence (TL) assessments. This comprehensive setup consisted of key components: an Inel XRG3500 X-ray generator equipped with a copper anode tube, operating at 45 kV/10 mA, and an Acton Research Corp. SpectraPro-500i monochromator, a Hamamatsu R928 photomultiplier tube (PMT), and an APD Cryogenic Inc. closed-cycle helium cooler. Initiating our investigation, we conducted afterglow measurements at a low temperature of 10 K by exposing the crystals to X-rays for a duration of 10 min. Subsequently, we recorded TL glow curves across a temperature spectrum spanning from 10 to 350 K, employing a heating rate of 9 K/min. In continuation, RL spectra were captured at various temperatures ranging from 350 to 10 K with intervals of 10 K, commencing from the highest temperature and progressing toward the lowest. This sequential approach was adopted deliberately to mitigate potential contributions arising from the thermal release of charge carriers to the overall emission yield.

*Time-Resolved RL.* Time-resolved radioluminescence (TRRL) spectra were acquired employing the time-correlated single photon counting method with a Start:Stop ratio of 5000:1, respectively. For generating X-ray pulses, a PicoQuant LDHP-C-440M pulsed diode laser was utilized, originating from a Hamamatsu N5084 X-ray tube stimulated by light, possessing an average energy of 18.2 keV. The laser activation was facilitated by a PicoQuant laser driver, where its reference output served as the start signal and was linked to an Ortec 567 time-to-amplitude converter (TAC). Precision in timing was ensured through the utilization of an Ortec 462 time calibrator employed to calibrate the bin width. The emitted photons were captured by an ID Quantique id100-50 single-photon counter, serving as the stop signal. Subsequent signal processing involved passing through a LeCroy 623B octal discriminator and analog delay. Time differences were then digitized by utilizing an Ortec AD114 amplitude to digital converter. The sample positioning was within a closed-cycle helium cryostat operating under pressures below 10^–4^ mbar, maintaining optimal conditions for experimentation.

*Computational Methods.* The band structure and energies of cubic and orthorhombic MAPbBr_3_ were calculated using density functional theory (DFT) as implemented in the Quantum Espresso package using the Perdew–Burke–Ernzerhof (PBEsol) exchange–correlation functional for solids. The kinetic energy cut off was set to 500 eV, and a 5 × 5 × 5 **k**-point mesh was used for sampling the Brillouin zone along the path Γ → M → X → R → Γ for the cubic unit cell and equivalent path Γ → S → Y → R → Γ for the orthorhombic unit cell. The convergence calculations were continued until the residual forces on the atoms were less than 0.01 eV/Å.

## Supporting Information Available

Synthesis details, XRD measurements, DFT calculations, glow curve, pulse height spectra, reflectance and dependent transmittance of Control-1M and THF-0.4M MAPbBr_3_ single crystals (PDF)
