# Supplementary material for: Improvement of Light Output of MAPbBr3 Single Crystal for Ultrafast and Bright Cryogenic Scintillator
Source: J Phys Chem Lett. 2024 Mar 28;15(14):3713–20. doi: 10.1021/acs.jpclett.4c00379 (PMC11017313; doi:10.1021/acs.jpclett.4c00379)
Supplement: Supplementary file 1 — jz4c00379_si_001.pdf [file jz4c00379_si_001.pdf]

Supporting Information for

Improvement of Light Output of $MAPbBr_3$ Single Crystal for Ultra-Fast and Bright Cryogenic Scintillator

Somnath Mahato[a,*], Michal Makowski[a,#], Shaona Bose[b], Dominik Kowal[a], Md Abdul Kuddus Sheikh[a], Philipp Braueninger-Wemer[c], Marcin E. Witkowski[d], Samit Kumar Ray[b], Winicjusz Drozdowski[d], Muhammad Danang Birowosuto[a,*]

[a]*Lukasiewicz Research Network - PORT Polish Center for Technology Development, Wroclaw, 54-066, Poland*

[b]*Department of Physics, Indian Institute of Technology Kharagpur, Kharagpur- 721302, India*

[c]*Cintilight LLC, Knoxville, TN 37919, USA*

[d]*Institute of Physics, Faculty of Physics, Astronomy, and Informatics, Nicolaus Copernicus University in Torun, Torun, 87-100, Poland*

[#]*On leave from Nicolaus Copernicus University in Torun, Torun, 87-100, Poland*

[*]*Corresponding author*

Synthesis details:

Chemicals: Lead bromide (98.99%), *N*-methylformamide (anhydrous, 99.8%), hydrobromic acid (99.98%) and tetrahydrofuran (99.99%) were purchased from Sigma Aldrich.

***Synthesis of Control-1M*:** One molar (1M) solution containing 1.101 gm of $PbBr_2$ was dissolved in 3ml of NMF and HBr solution with the ratio of 5.7:1 at room temperature. The solution was ultrasonicated for 30 minutes to dissolve properly then separated into three bottles and each of them were 1ml solution. All procedures were carried out under ambient conditions and humidity of 55–60%.

***Synthesis of THF-0.4M*:** 0.4M molar (0.4M) solution containing 1.468 gm of $PbBr_2$ was dissolved in 10 mL of NMF and HBr solution with the ratio of 5.7:1 at room temperature. The solution was ultrasonicated for 15 minutes to dissolve properly. After that, 10 mL of the respective precursor solutions was transferred in 90 mm small crystallization dish. The crystallization dish was wrapped by aluminum foil and a small hole in the center of the foil was made using a needle.

Finally, the small 40 mm small crystallization dish was dip into 150 mm of another big crystallization dish with 80 ml of THF. Perforating the aluminum foil allows the initialization of the crystallization by the volatile THF, which is a commonly used antisolvent for perovskite materials. The crystals of $MAPbBr_3$ were collected after 1–2 days on filter paper and dried for 24 h in a vacuum oven kept at 40 °C.

Experimental details:

Pulse height spectra: In order to acquire pulse height spectra using Am^{241} gamma excitation under low-temperature conditions, a sample was meticulously positioned at the base of a concave stainless-steel container designed in a parabolic shape. This container was firmly attached to the cryostat's cold finger, which was filled with liquid nitrogen. The container's structure was specifically devised to guide the scintillation light produced by the sample through a designated window, redirecting it toward a photomultiplier tube (XP2020Q, biased with -2300 V) positioned outside the cryostat. This setup was meticulously arranged to ensure the efficient detection of almost all emitted scintillation light. For the purpose of conducting pulse height measurements, the output signal from the photomultiplier tube (PMT) underwent processing via a custom-made preamplifier and further shaping using an Ortec 572 spectroscopic amplifier. Through these measurements, we were able to establish the relative scintillation yield as a variable dependent on temperature. To ascertain the photon yield per mega electronvolt of absorbed gamma-ray energy (ph/MeV) at room temperature, the methodology outlined by Maddalena et al. (1) was employed as a reference.

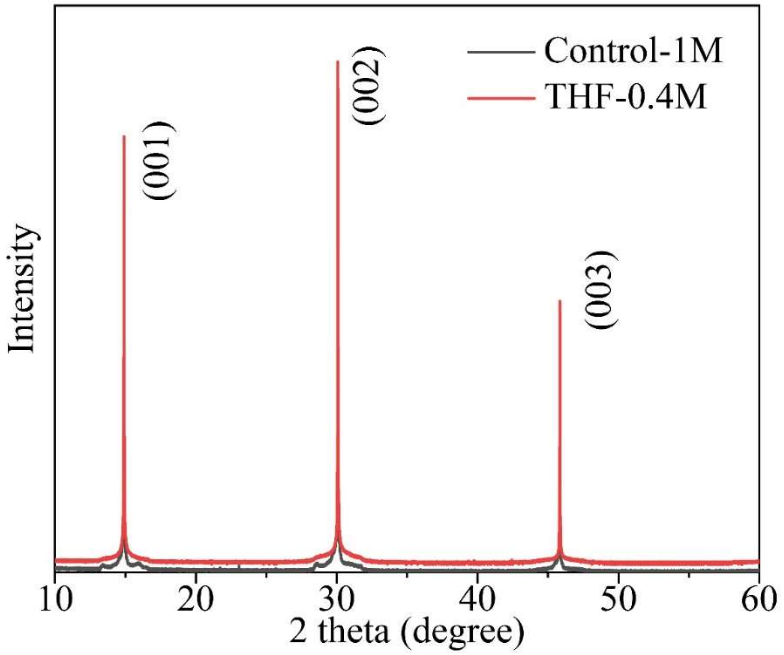

Figure S1*: XRD pattern of the Control-1M and THF-0.4M $MAPbBr_3$ single crystals.*

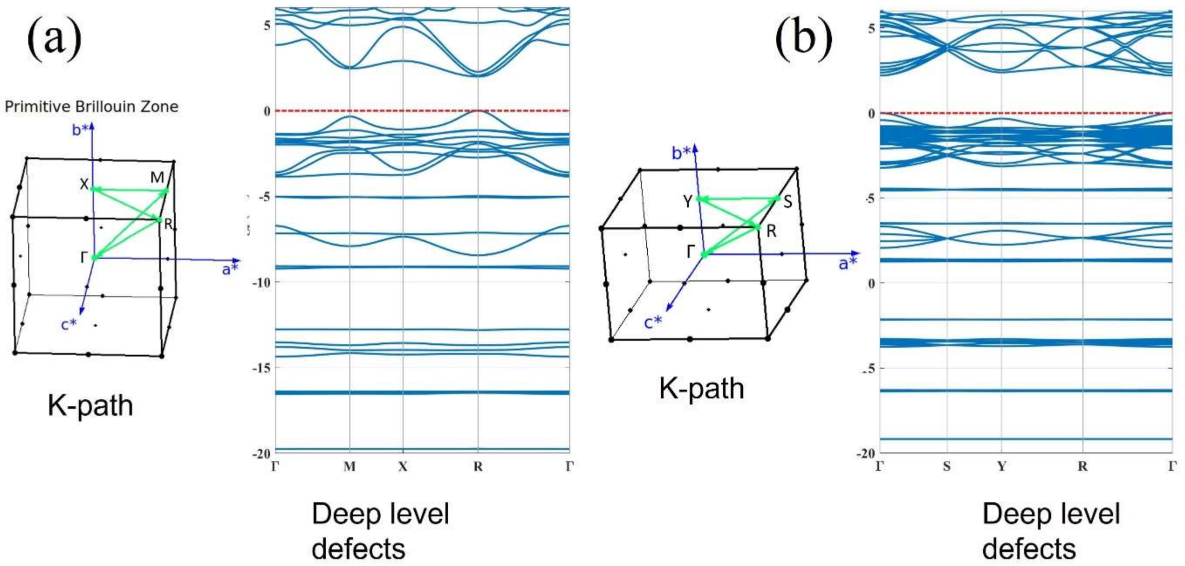

Figure S2: *Deep level defects of (a) cubic and (b) orthorhombic phase of* $MAPbBr_3$

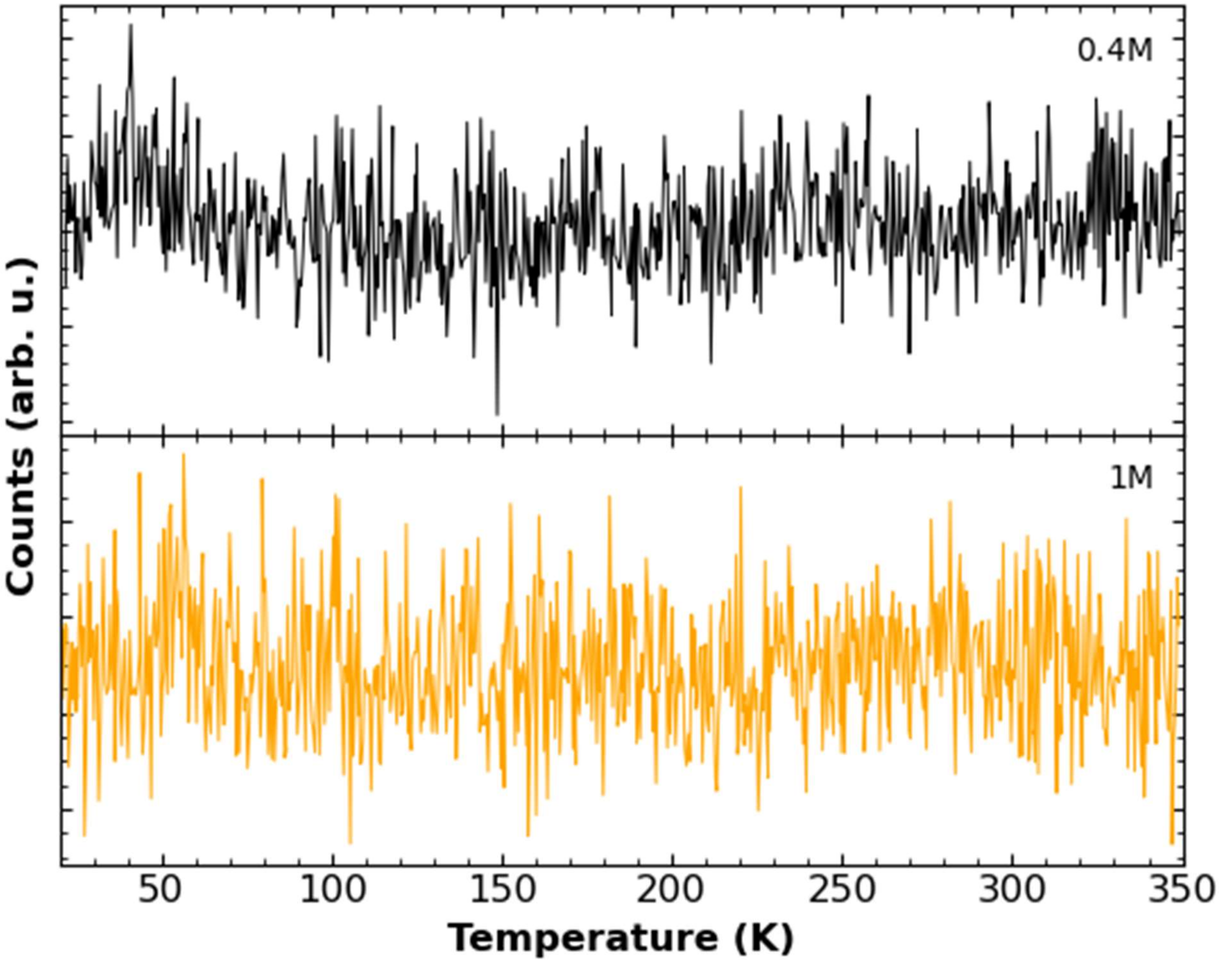

Figure S3: *Low-temperature glow curves of THF-0.4M (black line) and Control-1M (orange line) $MAPbBr_3$ single crystals*

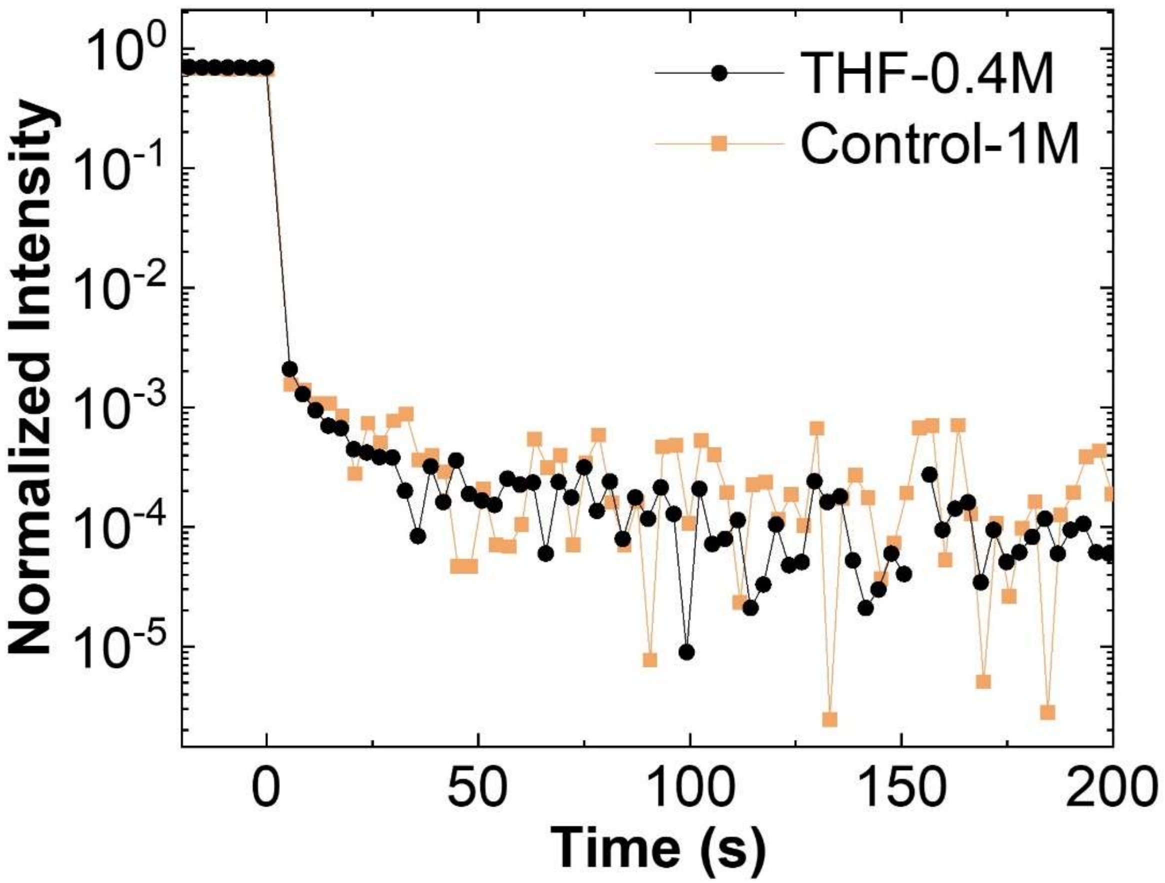

Figure S4: *X-ray excited afterglow of THF-0.4M (black line) and Control-1M (orange line) $MAPbBr_3$ single crystals*

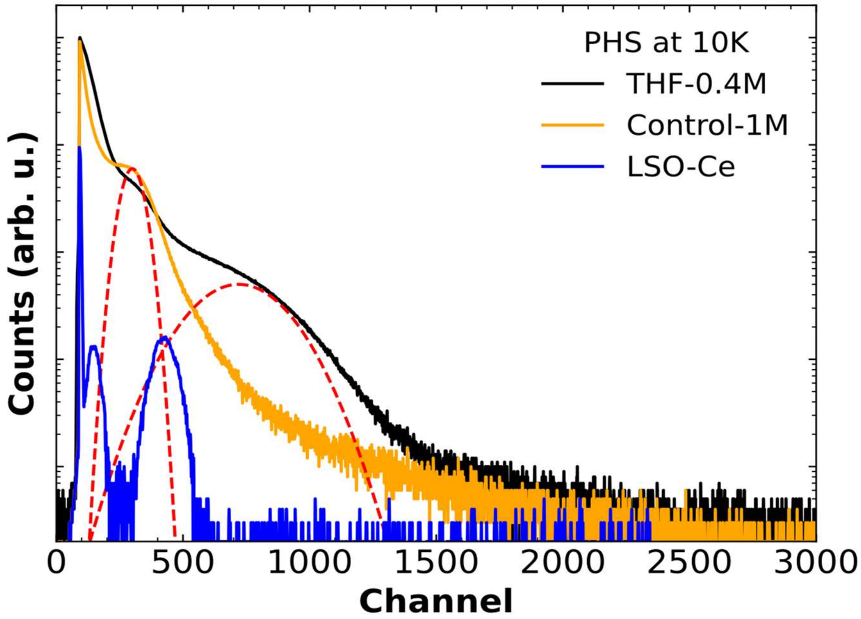

Figure S5: *Pulse height spectra of Am^{241} at 10K.*

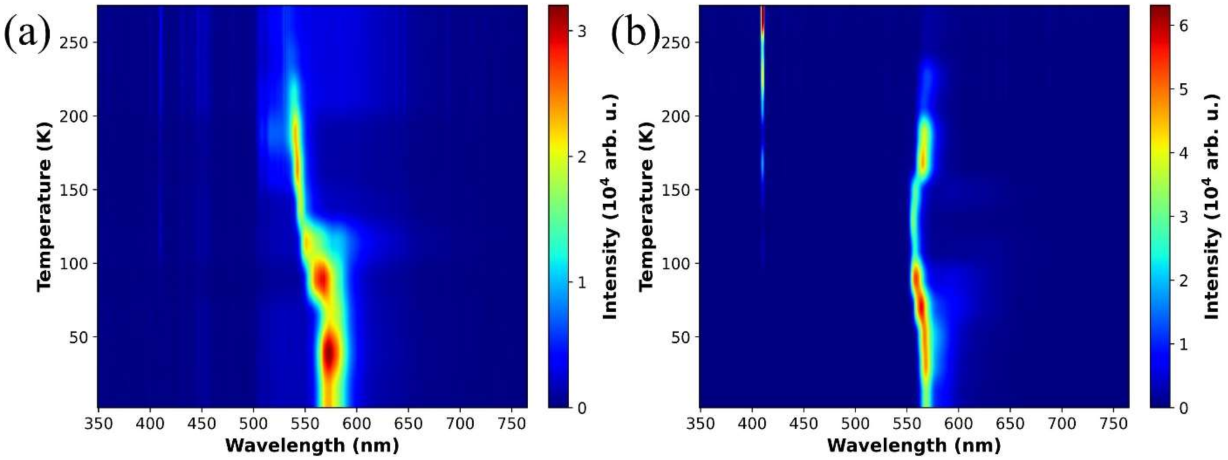

Figure S6: *Temperature dependent reflectance of (a) Control-1M and (b)THF-0.4M $MAPbBr_3$ single crystals*

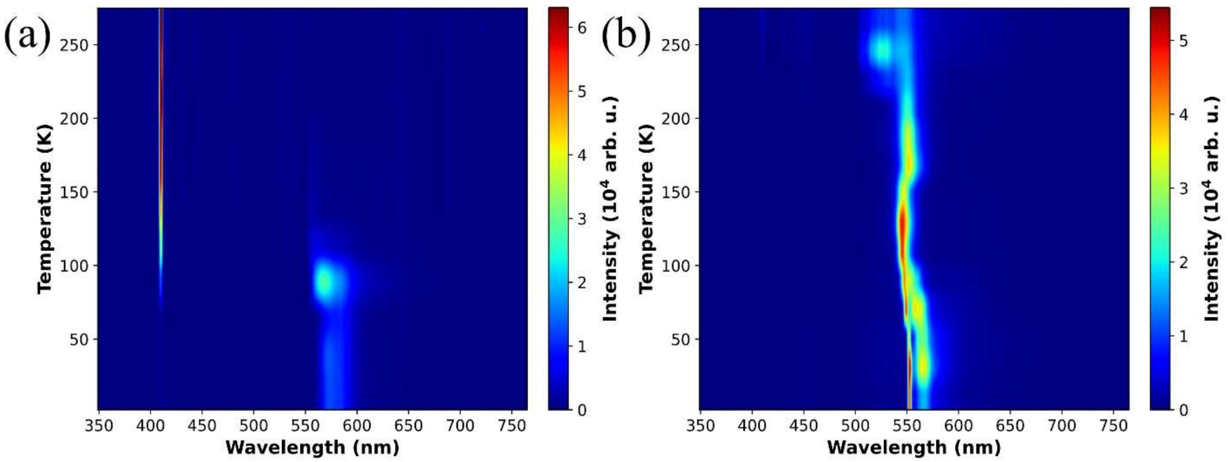

Figure S7: *Temperature dependent transmittance of (a) Control-1M and (b)THF-0.4M $MAPbBr_3$ single crystals*
